# Effect of preoperative intranasal insulin on postoperative delirium in adult surgical patients: a systematic review, meta-analysis, and trial sequential analysis

**DOI:** 10.1186/s13741-026-00674-5

**Published:** 2026-03-20

**Authors:** Emmanuel Mark M. Velasco, Vinicius Sepúlveda Lima, Tulio Caldonazo, Felipe S. Passos, Paulo Ricardo Gessolo Lins, Iago Teles Costa Grillo, Pedro Tanaka, Alex Macario, Aleksandar R. Zivkovic, João da ManoelSilva, Johannes Ehler, Ricardo Esper Treml

**Affiliations:** 1 Department of Anesthesiology and Pain Medicine, Toronto General Hospital, Toronto, Canada; 2 Centro de Ensino E Treinamento Do Hospital Português - Clínica de Anestesia de Salvador, Salvador, Brazil; 3 Department of Cardiothoracic Surgery, Friedrich-Schiller-University, Jena, Germany; 4 Departament of Thoracic Surgery, MaterDei Hospital, Salvador, Brazil; 5 Disciplina de Medicina de Urgência E Medicina, Baseada Em Evidências- Escola Paulista de Medicina-Universidade Federal de São Paulo, São Paulo, Brazil; 6 University of Buenos Aires, Buenos Aires, Argentina; 7 Department of Anesthesiology, Perioperative and Pain Medicine, Stanford School of Medicine, 300 Pasteur Dr Rm H3577MC 5640, Stanford, CA 94305 USA; 8 Medical Faculty Heidelberg, Department of Anesthesiology, Heidelberg University, Heidelberg, Germany; 9 Postgraduate in Anesthesiology, Surgical Sciences and Perioperative Medicine, University of São Paulo, São Paulo, Brazil; 10 Department of Anesthesiology and Intensive Care Medicine, Friedrich-Schiller-University, Jena, Germany

**Keywords:** Intranasal insulin, Postoperative delirium, Neuroinflammation, Perioperative neuroprotection

## Abstract

**Background:**

Postoperative delirium (POD) is a frequent and serious complication, especially in older adults and high-risk surgical patients. Intranasal insulin has emerged as a potential neuroprotective intervention, possibly modulating neuroinflammation and preserving cognitive function. This study aimed to evaluate the efficacy and safety of preoperative intranasal insulin in reducing POD incidence.

**Methods:**

A systematic review and meta-analysis were conducted according to PRISMA guidelines. PubMed, Embase, and the Cochrane Library were searched through March 2025 for randomized controlled trials (RCTs) comparing intranasal insulin versus placebo in adult surgical patients. The primary outcome was the incidence of POD. Secondary outcomes included inflammatory biomarkers (IL-6, TNF-α, CRP), glucose levels, and insulin resistance (HOMA-IR). Odds ratios (OR) and mean differences (MD) with 95% confidence intervals (CI) were calculated using random-effects models. Trial Sequential Analysis (TSA) was also performed.

**Results:**

Eight RCTs from China involving 888 patients (481 intranasal insulin, 407 placebo) were included. Intranasal insulin significantly reduced the risk of POD (OR 0.24; 95% CI 0.17 to 0.34; *p* < 0.001; I^2^ = 0%). TSA confirmed the robustness of this finding. Significant reductions in IL-6 (MD -2.52; 95% CI -3.45 to -1.58) and TNF-α (MD -5.02; 95% CI -8.67 to -1.36) were observed. No significant differences were found in serum or CSF glucose, or HOMA-IR. No hypoglycemia or serious adverse events were reported.

**Conclusion:**

Preoperative intranasal insulin is a safe and effective strategy to reduce POD, likely through central anti-inflammatory mechanisms, without compromising glycemic stability.

**Graphical Abstract:**

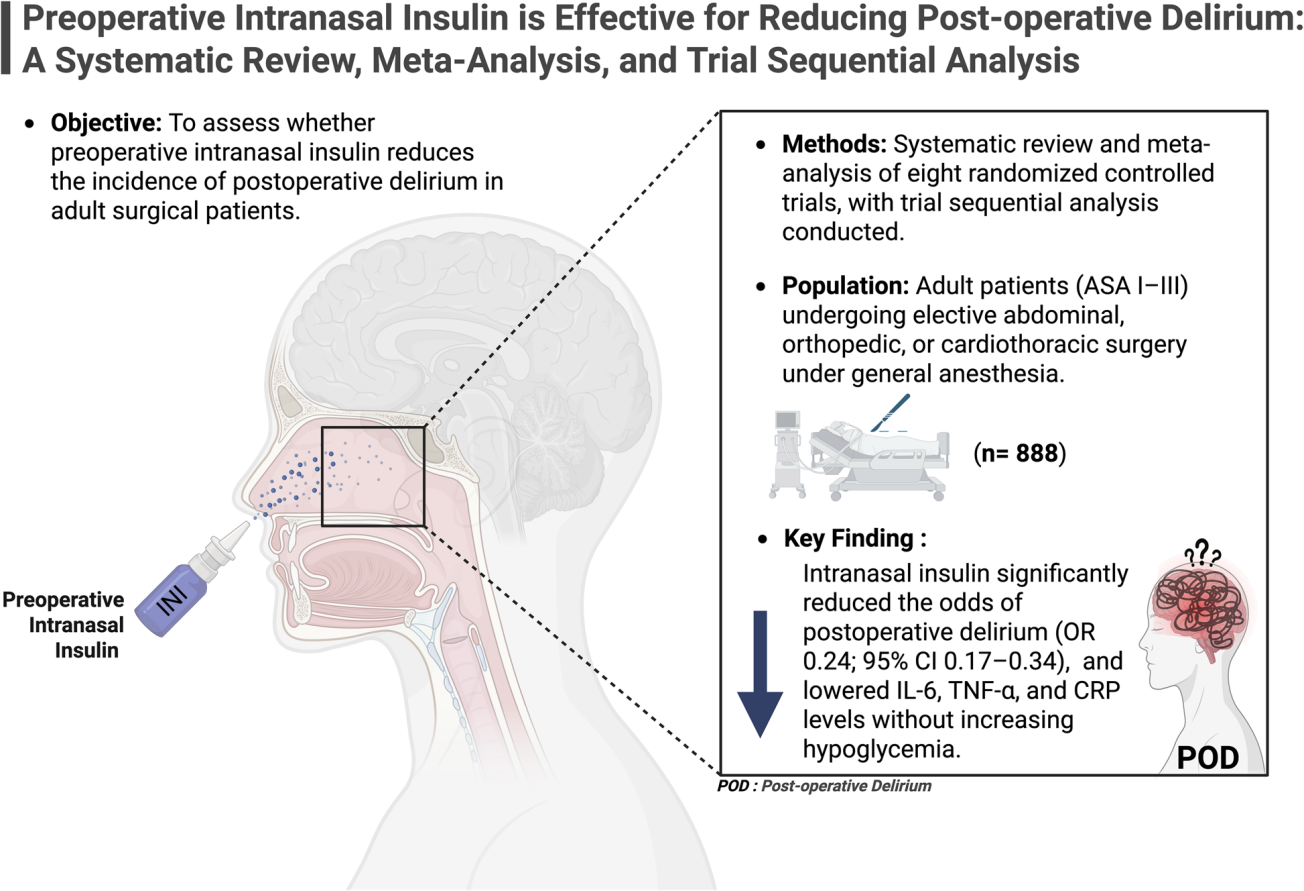

**Supplementary Information:**

The online version contains supplementary material available at 10.1186/s13741-026-00674-5.

## Key points summary

Question: Is preoperative intranasal insulin effective in reducing the incidence of postoperative delirium in adult surgical patients?

Findings: Intranasal insulin significantly reduced postoperative delirium and systemic inflammation without affecting glycemic stability.

Meaning: Preoperative intranasal insulin is a safe and promising strategy for perioperative neuroprotection in high-risk patients.

**Supplementary Information:**

The online version contains supplementary material available at 10.1186/s13741-026-00674-5.

## Introduction

Postoperative delirium (POD) represents a significant and complex complication in patients undergoing surgical procedures, particularly among older adults and those with pre-existing risk factors. (Inouye et al. 2014) It is characterized by an acute and fluctuating disturbance in attention, awareness, and cognition, and is associated with prolonged hospitalization, increased healthcare costs, and elevated risks of long term cognitive decline and mortality (Inouye et al. 2014). Despite advances in perioperative care, effective preventive strategies remain limited. (Aldecoa et al. 2017)

The pathophysiology of POD is not completely understood; however, a multifactorial pathway has been proposed (Inouye et al. 2014). Surgical stress triggers a systemic inflammatory response, leading to increased circulating cytokines that may disrupt blood brain barrier integrity and impair neuronal signaling and metabolism (Maldonado 2013; Saxena and Maze 2018). This neuroinflammatory cascade is increasingly recognized as a major contributor to cognitive dysfunction in delirium and represents a potential therapeutic target. (Xiao et al. 2023)

Insulin, traditionally known for its metabolic role, has emerged as a candidate neuroprotective agent.⁶ Intranasal delivery bypasses the blood brain barrier and permits direct central nervous system engagement. (Badenes et al. 2021) Intranasal insulin likely exerts its protective effects through central modulation of neuroinflammation, rather than systemic cytokine suppression. Elevated IL-6, TNF-α, and CRP are associated with increased blood–brain barrier permeability, neuroinflammation, and cognitive dysfunction (Sun et al. 2023; Li et al. 2019). When administered via the intranasal route, insulin circumvents the blood–brain barrier and directly influences central nervous system function. (Badenes et al. 2021) Intranasal insulin exerts neuroprotective effects by preserving cerebral glucose metabolism (Yu and Pei 2015), stabilizing neurotransmission (Canteiro et al. 2019), and attenuating central inflammatory cascades triggered by cytokine surges (Canteiro et al. 2019; Qeva et al. 2022).

Randomized clinical trials evaluating perioperative intranasal insulin have reported a 40% to 60% reduction in POD incidence across cardiac, orthopedic, and abdominal surgeries (Huang et al. 2021, 2023, 2024; Liu et al. 2025a; Mi et al. 2023; Zhang et al. 2024). However, existing pharmacologic reviews of POD prevention have not examined intranasal insulin, leaving an important knowledge gap. (Park et al. 2021) This systematic review, meta-analysis, and trial sequential analysis (TSA) aimed to determine whether intranasal insulin can reduce the incidence of postoperative delirium and modulate associated inflammatory biomarkers.

## Methods

This systematic review and meta-analysis was conducted in accordance with the methodological guidance outlined by the Cochrane Handbook for Systematic Reviews of Interventions and was reported in compliance with the Preferred Reporting Items for Systematic Reviews and Meta-Analyses (PRISMA) statement. (Moher et al. 2009) The review was registered in the International Prospective Register of Systematic Reviews (PROSPERO), under protocol number CRD42025643975 (EMMV Jr. 2025).

### Search strategy

A systematic literature search was performed on Embase, PubMed, and the Cochrane Library from inception through March 2025 using the following search terms: “intranasal insulin”, “central insulin administration”, “delirium”, “cognitive”, and “memory”. The complete search strategy is detailed in Supplementary Table 1. No language restrictions were applied. In addition, the reference lists of all included articles, as well as prior systematic reviews and meta-analyses, were manually screened to identify any additional eligible studies.

### Study selection

Two independent reviewers (EMV and VSL) screened the records, after deduplication. Any discrepancies and disagreements were resolved by consensus. Titles and abstracts were reviewed against pre-defined inclusion and exclusion criteria.

### Eligibility criteria

We applied prespecified eligibility criteria to ensure methodological rigor and minimize selection bias. Eligible studies were randomized controlled trials enrolling adults aged 18 years or older undergoing elective or emergency surgery under general anesthesia. Surgical categories permitted included abdominal, orthopedic, vascular, thoracic, and mixed non-cardiac procedures. Trials were required to enroll patients classified as ASA physical status I–III; to administer intranasal insulin via spray or instillation; to use intranasal normal saline as the comparator with identical dosing frequency; and to report POD incidence as a predefined outcome. A minimum postoperative follow-up of 3 days was required to ensure consistent ascertainment of POD.

Studies were excluded if they used nonrandomized or quasi-experimental designs; if they were observational studies, reviews, case reports, conference abstracts, or animal studies; or if they provided incomplete or inaccessible outcome data that precluded quantitative synthesis. Trials conducted exclusively under regional or neuraxial anesthesia, studies lacking a placebo or control arm, and those that did not measure or report POD incidence were also excluded. Publications in languages other than English were not considered.

Because three studies originated from the same research group, we implemented predefined rules to avoid overlapping populations: only trials enrolling distinct surgical cohorts were retained, and the dataset with the most complete, nonredundant information was included. Studies administering insulin through routes other than intranasal delivery were likewise excluded.

### Risk of bias and publication bias assessment

Risk of bias for each included study was independently assessed by two reviewers (EMV and VS), with any discrepancies adjudicated by a third reviewer (VS). The Cochrane Collaboration’s Risk of Bias 2 (RoB-2) tool was utilized to evaluate randomized trials across five domains: bias arising from the randomization process, bias due to deviations from intended interventions, bias due to missing outcome data, bias in the measurement of the outcome, and bias in the selection of the reported result. Publication bias was qualitatively assessed through visual inspection of funnel plot symmetry for the primary outcome. We also applied a prespecified strategy for managing studies originating from the same institution. Although three eligible trials were published by the same research group, a detailed assessment confirmed that each study enrolled a distinct surgical cohort with no overlap in participants. These cohorts represented three separate clinical populations and were therefore included as independent datasets. This approach prevented duplication of patient data while allowing incorporation of all methodologically eligible evidence. Given the limited number of studies in this meta-analysis, no formal quantitative assessment for small-study effects (e.g., Egger’s test) was performed.

### Data extraction

Two reviewers (EMV and VS) independently extracted data using a standardized data collection form. The following variables were retrieved from each eligible study: study characteristics (first author, publication year, sample size, and type of surgery), patient baseline characteristics (age, sex, population profile, and ASA physical status), details of the intervention (intranasal insulin regimen), control group characteristics (placebo), laboratory data (in particular perioperative glucose levels), and reported outcome measures.

### Endpoints and subgroups analysis

The primary outcome was the incidence rate of postoperative delirium. The secondary outcomes were serum levels of interleukin-6 (IL-6), tumor necrosis factor-alpha (TNF-α), C-reactive protein (CRP), and glucose, cerebrospinal fluid (CSF) levels of glucose, and the homeostatic model assessment of insulin resistance (HOMA-IR) index.

The incidence of delirium was assessed based on the use of different scales in the individual trials: Confusion Assessment Method for the Intensive Care Unit (CAM-ICU), (Miranda et al. 2023) Revised Delirium Rating Scale (DRS-R98), (Almuhairi et al. 2024) 3-min Diagnostic assessment for CAM-defined delirium (3D-CAM), (Marcantonio et al. 2014) International Study of Postoperative Cognitive Dysfunction (ISPOCD). (Moller et al. 1998) The study by Mi (Huang et al. 2023) used a battery of neuropsychological tests, namely, the verbal learning and fluency test, the visuospatial memory and delayed recall test, the Benton judgment of line orientation, the trail-making test parts A and B, the digit span test, and the digit symbol substitution test.

### Statistical analysis

For binary outcomes, risk ratios (RRs) with 95% confidence intervals (CIs) were estimated, whereas continuous outcomes were summarized as mean differences (MDs) with 95% CIs. Random-effects models according to the DerSimonian and Laird method were applied for all endpoints, given the anticipated heterogeneity in study methodologies and participant demographics. (Schandelmaier et al. 2020) Heterogeneity was assessed using the I^2^ statistic and Cochran’s Q test, with *p*-values < 0.10 and I^2^ values > 25% considered indicative of significant heterogeneity. Subgroup analyses evaluating potential heterogeneity introduced by dexmedetomidine use in some trials were performed.

To address the robustness of the findings, a leave-one-out analysis was performed for the primary outcome. (Deeks et al. 2019) The Cochrane Handbook for Systematic Reviews of Interventions was used for data handling and conversion.

All statistical analyses were performed using Software R, version 4.4.0 (R Foundation for Statistical Computing, Vienna, Austria). All data extracted from graphs were obtained using WebPlotDigitizer.

### Trial sequential analysis (TSA)

A TSA was conducted for the primary outcome to determine whether the cumulative evidence was adequately powered to reach a definitive conclusion. The statistical plan prespecified two-sided testing with a type I error rate of 5% and a type II error rate of 20%, corresponding to 80% statistical power. Both conventional and trial sequential monitoring boundaries (TSMBs) were generated for the intranasal insulin and placebo groups. Heterogeneity adjustment was based on a variance-based correction method, and a random-effects model was applied throughout the sequential analyses. A cumulative z-curve was constructed to assess the sufficiency and robustness of the available evidence relative to the monitoring boundaries. Additionally, the required information size (RIS)—the total sample size needed to confirm or refute the intervention effect with adequate statistical power—was calculated. By definition, TSA provides stronger inferential validity when the accrued sample size exceeds the calculated RIS, minimizing the risks of type I and type II errors inherent in cumulative meta-analyses.

## Results

### Study selection and characteristics

**Fig. 1:**
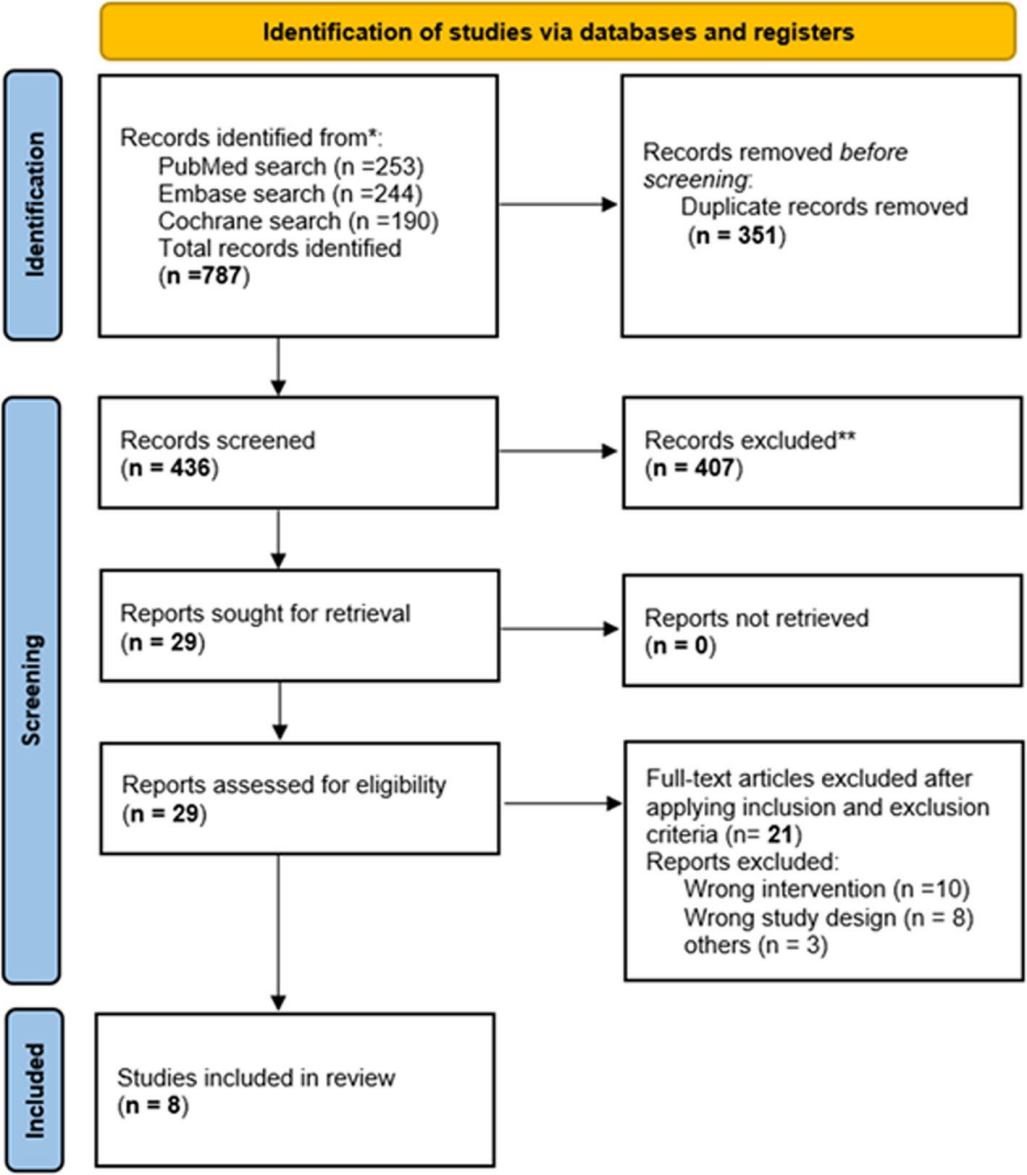
Preferred Reporting Items for Systematic Reviews and Meta-Analyses (PRISMA) flow diagram

Figure 1 shows the PRISMA flow diagram outlining the study selection process. The initial search, conducted in March 2025, yielded a total of 787 records. After removal of duplicates and application of eligibility and exclusion criteria, 29 studies were selected for full-text review. Of these, 8 studies were included in the final analysis (Huang et al. 2021, 2023, 2024; Mi et al. 2023; Zhang et al. 2024; Yang et al. 2025; Sun et al. 2024; Li et al. 2025) The primary reasons for exclusion were studies presenting only protocols and investigations evaluating alternative interventions. During study selection, we noted that three publications from the same research group (Huang et al. 2021, 2023, 2024) reported coinciding features, including identical ethics approval numbers, similar trial registry details, and recruitment during comparable time periods. As these reports may have originated from the same underlying surgical patient pooling, we first conducted the analysis including all three trials. Subsequently, a sensitivity analysis excluding these studies was performed to assess the robustness of the observed effect and to mitigate the potential bias from overlapping populations. Their characteristics are presented for completeness, and the results of both analyses are provided in the Supplementary Appendix.

### Risk of bias and publication bias assessment

According to the RoB2 assessment, six randomized controlled trials were judged to have low risk of bias (Huang et al. 2021, 2023, 2024; Mi et al. 2023; Zhang et al. 2024; Li et al. 2025), one was rated as having some concerns, (Yang et al. 2025) and one was identified as high risk. (Sun et al. 2024) of bias (Supplementary Fig. 1). The factors contributing to higher risk classifications primarily involved issues in the randomization process and limitations in outcome assessment Visual inspection of funnel plots demonstrated no evidence of small study effects or publication bias for the primary outcome. (Supplementary Fig. 2).

### Patient characteristics

Table 1 shows the individual study information. Table 2 presents the insulin protocols and delirium evaluation methods across studies. A total of eight RCTs were included in this meta-analysis, encompassing 888 patients. Among these, 481 (54%) received intranasal insulin therapy. The mean age of participants across studies ranged from 53 to 82 years, and the proportion of male patients varied from 24 to 69%. The surgical procedures included cardiac, gastrointestinal, and orthopedic surgeries.

**Table 1:** Study and patient’s baseline characteristics of included studies

**Study**	**Surgery type**	**Sample size; INI/Placebo (n)**	**Age (years), INI/Placebo**	**Male INI/Placebo, n**	**BMI (kg/m**^**2**^**); INI/Placebo**	**Duration of Surgery (min); INI/Placebo**	**Blood loss**, (**ml); INI/Placebo**	**Baseline MMSE score; INI/Placebo**
Huang et al. 2021	Laparoscopic gastrointestinal surgery	40/40	68/65	63/53	21.6/20.9	NR	NR	NR
Huang et al. 2023	Esophageal surgery	60/30	68/68.5	57/53	22.6/23.6	260.25/247.6	267.5/225	19.25/19
Huang et al. 2024	Cardiac valve surgery	36/35	53/55	33.3/40	23.3/22.3	256.6/254.6	600/600	26.9/26.7
Li et al. 2025	Orthopedic Hip arthroplastic surgery	87/43	80.9/82	28.7/27.9	20.7/20.7	74.2/73.8	NR	NR
Mi et al. 2023	Non-cardiac surgeries	58/58	69.8/70.6	51/59	NR	215.3/228.9	234/234	25/25.5
Yang et al. 2025	Orthopedic arthroplastic surgery	106/106	72.8/73	25/24	23.1/23.8	240.6/249	230.6/238.3	25.2/25.6
Sun et al. 2024	Orthopedic and pancreatic surgery	64/64	68.7/68.9	35/41	26.2/25.2	NR	NR	NR
Zhang et al. 2022	Laparoscopic colorectal surgery	30/31	70.7/69.9	63/69	22.1/21.6	216.6/213.5	NR	29.7/28.4

*BMI* Body Mass Index, *INI* Intranasal insulin, *MMSE* Mini-Mental State Examination, *NR* Not reported

**Table 2:** Insulin protocols, delirium evaluation, and follow-up across studies

Study	Surgery type	Insulin regimen and timing	Dexmedetomidine use?	Delirium evaluation method	Time of follow-up
Huang et al. 2021	Laparoscopic gastrointestinal surgery	20 U BID × 2 days + 1 dose pre-op	no	CAM-ICU	5 days
Huang et al. 2023	Esophageal surgery	20 U or 30 U BID × 2 days + 1 dose pre-op	no	CAM-ICU	3 days
Huang et al. 2024	Cardiac valve surgery	20 U BID × 2 days + 1 dose pre-op	yes	CAM-ICU	3 days
Li 2025	Orthopedic Hip arthroplastic surgery	20 U or 40 U × 3 doses	yes	CAM-ICU	3 days
Mi et al. 2023	Non-cardiac surgeries	40 U daily × 10 days	no	Various neuropsychologic tests*	7 days
Yang et al. 2025	Orthopedic arthroplastic surgery	40 U daily × 8 days	no	CAM-ICU + DRS	5 days
Sun et al. 2024	Orthopedic and pancreatic surgery	40 U daily × 4 days	no	3D-CAM	3 days
Zhang et al. 2022	Laparoscopic colorectal surgery	20 U BID × 2 days + 1 dose pre-op	no	ISPOCD	7 days

*CAM-ICU* Confusion Assessment Method for the Intensive Care Unit, *DRS* Revised Delirium Rating Scale, *3D-CAM* 3-min Diagnostic Assessment for CAM-defined delirium, *ISPOCD* International Study of Postoperative Cognitive Dysfunction, *U* Units

^*^Various neuropsychologic tests: verbal learning and fluency test, the visuospatial memory and delayed test, the Benton judgment of line orientation, the trail

### Primary outcome

#### Postoperative delirium

**Fig. 2:**
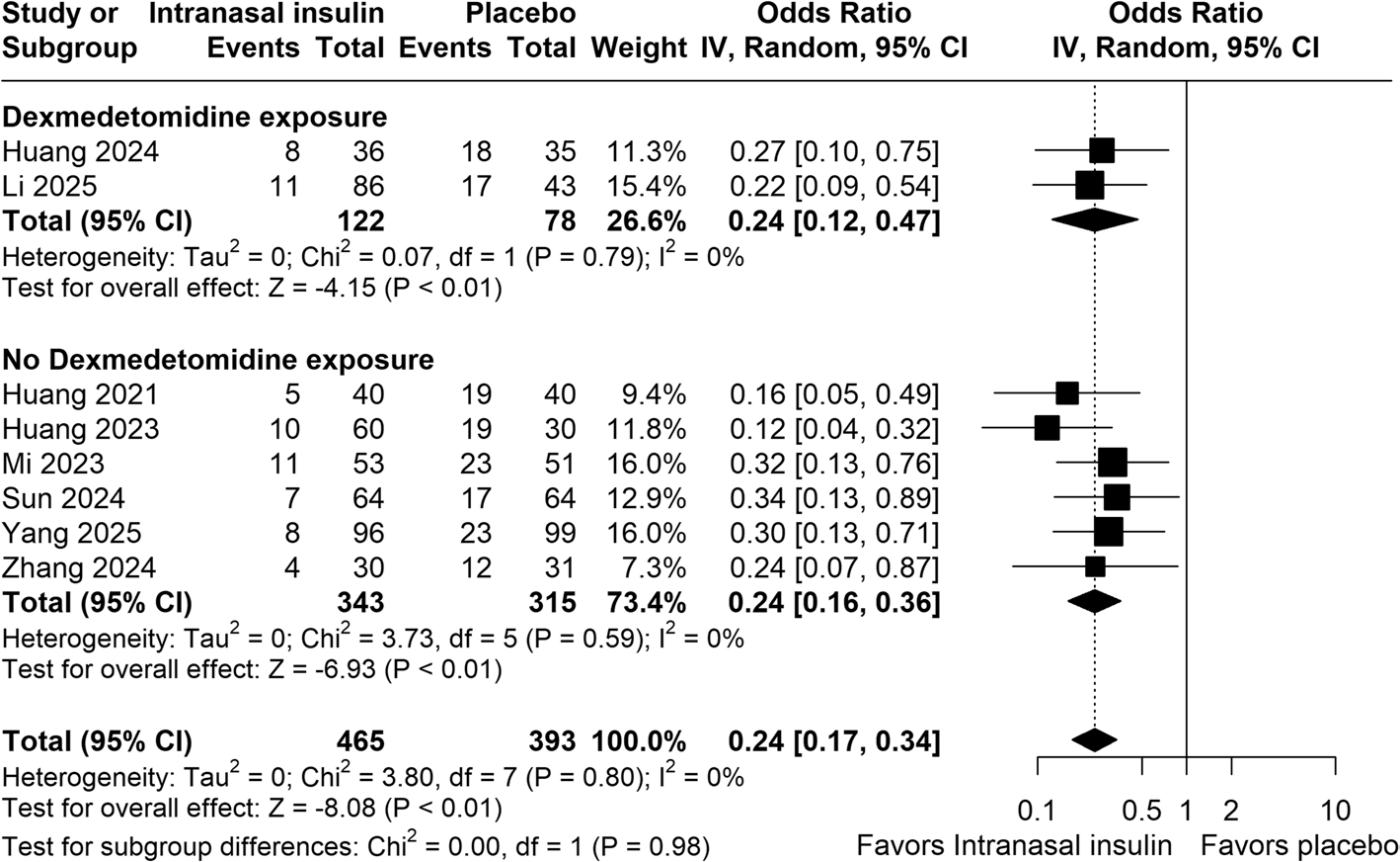
Forest plot comparing the effect of preoperative intranasal insulin on postoperative delirium and impact of dexmedetomidine co-exposure. INI: intranasal insulin; POD: postoperative delirium; CI: confidence interval; OR: Odds ratio

Table 3 summarizes the meta-analysis findings, highlighting a significant reduction in the incidence of postoperative delirium in the intranasal insulin group (OR 0.24; 95% CI 0.17 to 0.34; *p* < 0.001, I^2^ = 0%; Fig. 2). This outcome was assessed in eight studies. Subgroup analysis stratified by intraoperative dexmedetomidine exposure demonstrated an equivalent reduction in delirium incidence in both strata: patients not receiving dexmedetomidine (OR 0.24; 95% CI 0.16 to 0.36; *p* < 0.01; I^2^ = 0%; Fig. 2) and those receiving dexmedetomidine (OR 0.24; 95% CI 0.12 to 0.47; *p* < 0.01; I^2^ = 0%; Fig. 2), with no significant interaction between subgroups (P for subgroup differences = 0.98; Fig. 2). Three additional trials (Huang et al. 2021, 2023, 2024) were identified but showed overlapping features suggestive of a shared patient cohort. To examine the robustness of our findings, a sensitivity analysis excluding these studies was conducted, which consistently demonstrated a significant reduction in postoperative delirium (OR 0.29; 95% CI 0.19 to 0.43; *p* < 0.001; I^2^ = 0%). The corresponding forest plots are presented in Supplementary Fig. 6, confirming that the exclusion of these studies did not change the direction or significance of the effect. Across the included trials, postoperative delirium was assessed using different instruments including CAM ICU, DRS R98, 3D CAM, ISPOCD testing, and one extended neuropsychologic battery. Although these tools differ in diagnostic thresholds and sensitivity, particularly for hypoactive presentations, the pooled analysis showed no heterogeneity (I^2^ = 0%). In the pooled analysis (Supplementary Fig. 8), intranasal insulin was associated with a significantly lower incidence of delirium, with a risk ratio of 0.36 (95% CI, 0.27–0.46)**.**

**Table 3:** Summary of outcomes

Outcome	Number of Studies	Number of Patients	Effect Estimate, Random Model (95% CI, *p*-value)
Postoperative delirium	8	888	OR 0.24; 95% CI 0.17 to 0.34; *p* < 0.001*,* I^2^ = 0%
Serum IL-6 levels (pg/mL)	3	269	MD −2.52; 95% CI −3.45 to −1.58; *p* < 0.001; I^2^ = 0%
Serum TNF-α levels (pg/mL)	4	373	MD −5.02; 95% CI −8.67 to −1.36; *p* = 0.007; I^2^ = 87%
Serum CRP levels (mg/dL)	2	232	MD −1.47; 95% CI −2.61 to −0.33; *p* = 0.01; I^2^ = 0%
Insulin resistance (HOMA-IR index)	3	427	MD −0.41; 95% CI −1.00 to 0.19; *p* = 0.18; I^2^ = 57%
Serum glucose (mg/dL)	5	468	MD 0.05; 95% CI −0.11 to 0.21; *p* = 0.52; I^2^ = 8%
CSF glucose (mmol/L)	2	276	MD 1.24; 95% CI −0.61 to 3.10; *p* = 0.19; I^2^ = 99%

*MD* mean difference, *OR* Odds ratio, *CI* confidence interval, *IL-6* interleukin-6, *TNF-α* tumor necrosis factor-alpha, *CRP* C-reactive protein, *HOMA-IR* homeostatic model assessment of insulin resistance, *CSF* cerebrospinal fluid

### Sensitivity analyses

The leave-one-out analyses confirmed the robustness of the primary outcome (Supplementary Fig. 3). No single study, when excluded, altered the direction or statistical significance of the overall effect.

### Trial sequential analysis

The trial sequential analysis (TSA) indicated that the cumulative z-curve crossed both the conventional boundary and the required information size (RIS) threshold, confirming that sufficient evidence exists to support the benefit of intranasal insulin in reducing postoperative delirium compared with placebo. Detailed TSA graphs are presented in Supplementary Fig. 4.

### Secondary outcomes

#### Serum level of IL-6

**Fig. 3:**
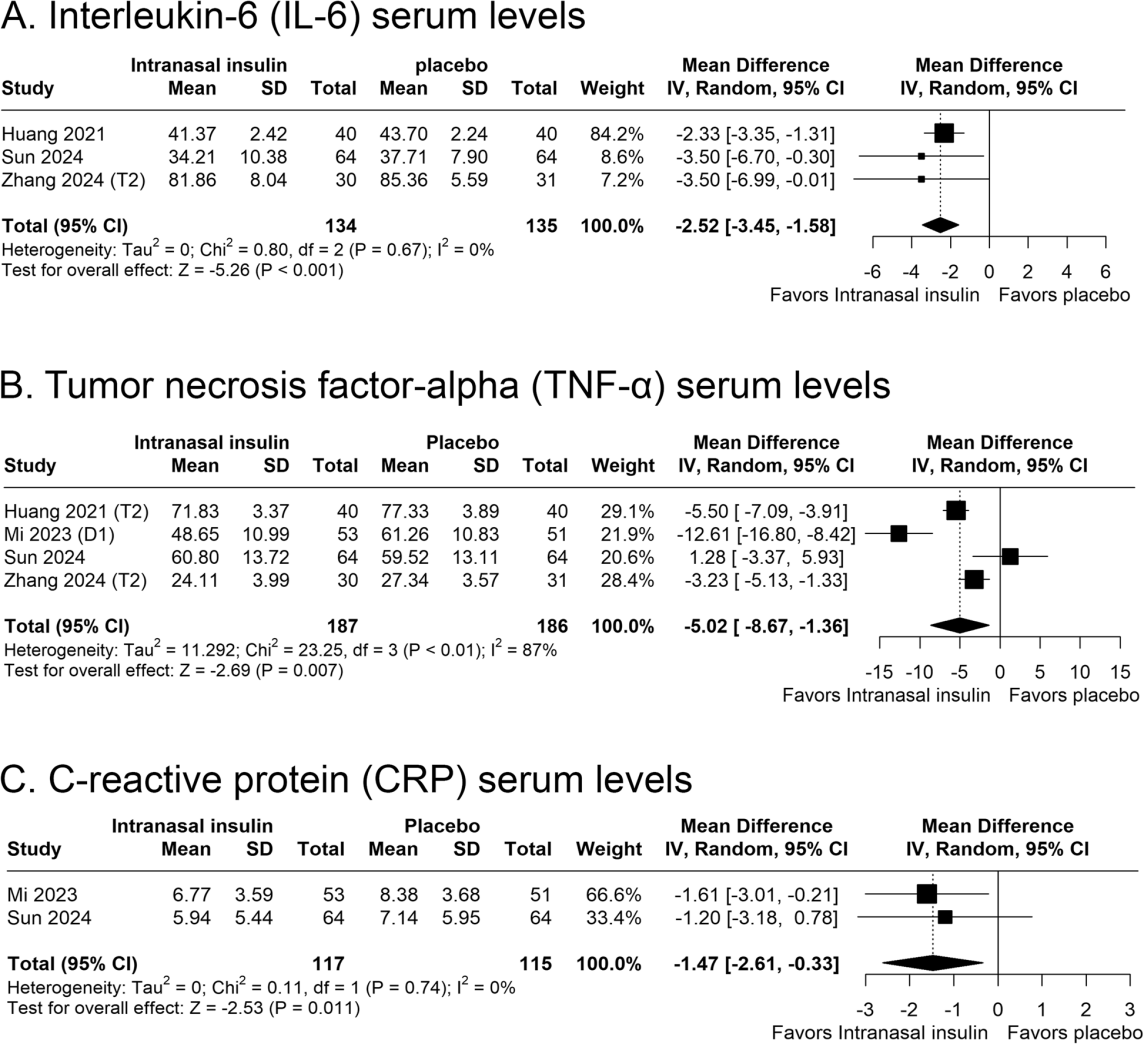
Forest plots presenting the pooled analyses of inflammatory biomarkers in patients receiving preoperative intranasal insulin versus placebo. **A** Pooled serum interleukin 6 (IL6) levels (pg/mL). **B** Pooled serum tumor necrosis factor alpha (TNFα) levels (pg/mL). **C** Pooled serum C reactive protein (CRP) levels (mg/L). CI, confidence interval; CRP, C reactive protein; IL6, interleukin 6; MD, mean difference; TNFα, tumor necrosis factor alpha

Three studies, encompassing 134 participants in the intranasal insulin group and 135 in the placebo group, reported postoperative IL-6 serum levels. Figure 3A shows a significant reduction in IL-6 concentrations was observed in patients receiving intranasal insulin compared to placebo (MD—2.52 pg/ml; 95% CI −3.45 to −1.58; *p* < 0.001; I^2^ = 0%; Fig. 3A).

#### Serum levels of TNF-α and CRP

Four studies, involving 373 patients, assessed TNF-α level outcome. Patients in the intranasal insulin group showed a significant reduction in TNF-α serum level, as demonstrated in Fig. 3B (MD −5.02 pg/ml; 95% CI −8.67 to −1.36; *p* = 0.007; I^2^ = 87%; Fig. 3B). Postoperative CRP serum level was reported in two studies. Intranasal insulin use was associated with a statistically significant reduction in CRP levels, as showed in Fig. 3C (MD −1.47 mg/dl; 95% CI −2.61 to −0.33; *p* = 0.01; I^2^ = 0%; Fig. 3C).

#### Serum and CSF Glucose levels and HOMA-IR index

**Fig. 4:**
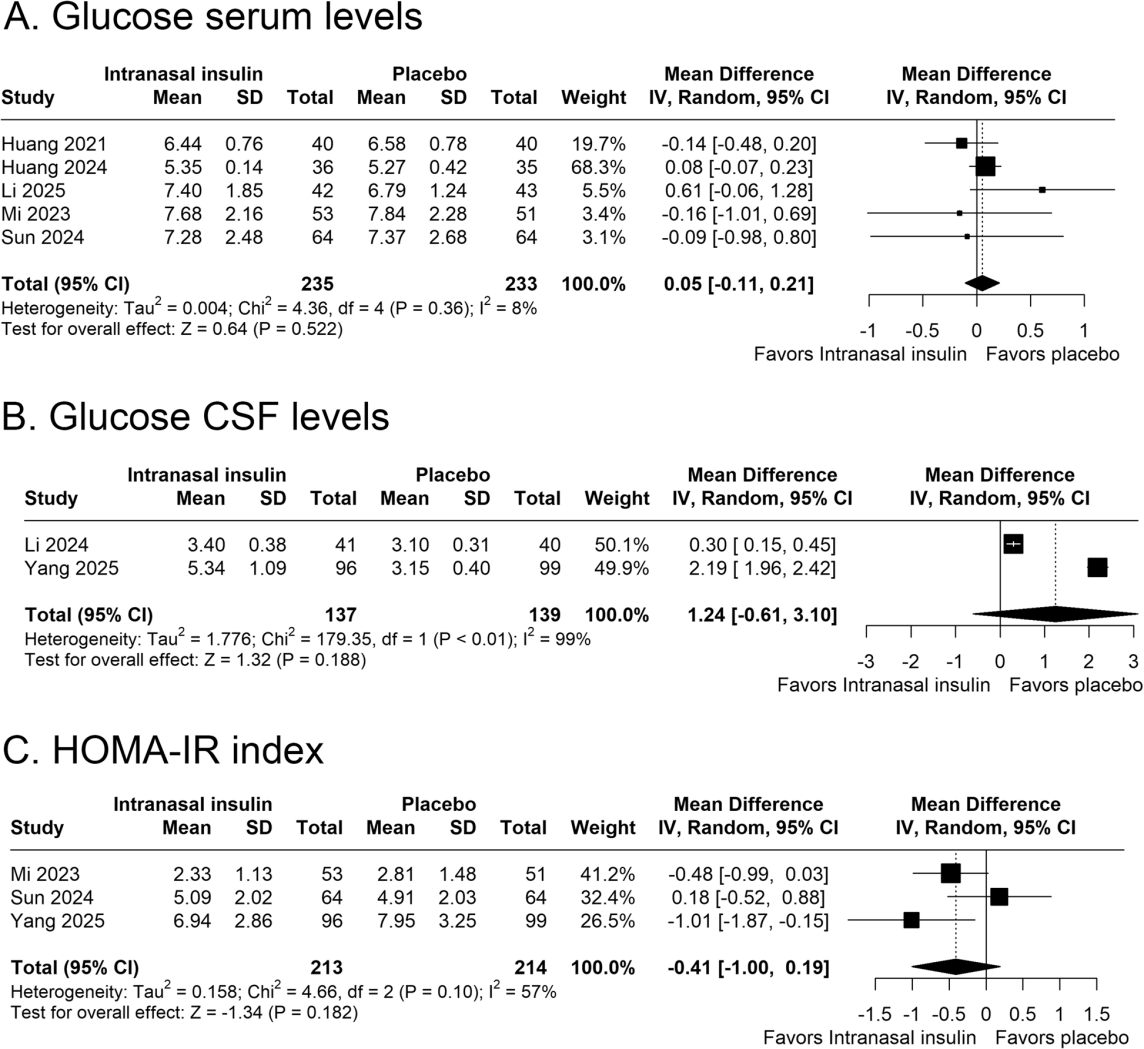
Forest plots presenting the pooled analyses of metabolic biomarkers in patients receiving preoperative intranasal insulin versus placebo. **A** Pooled serum glucose levels (mg/dL). **B** Pooled cerebrospinal fluid (CSF) glucose levels (mmol/L). **C** Pooled HOMA IR index. CI, confidence interval; CSF, cerebrospinal fluid; HOMA IR, homeostatic model assessment of insulin resistance; MD, mean difference; SD, standard deviation

Serum glucose levels, pooled from five studies, showed no statistically significant difference between intranasal insulin and placebo groups, as demonstrated in Fig. 4A (MD 0.05 mg/dL; 95% CI –0.11 to 0.21; *p* = 0.52; I^2^ = 8%; Fig. 4A).

Figure 4B evidences the pooled analysis for postoperative CSF glucose concentrations, assessed in two studies. There was no significant difference between groups (MD 1.24 mmol/L; 95% CI –0.61 to 3.10; *p* = 0.19; I^2^ = 99%; Fig. 4B).

Postoperative HOMA-IR index was reported in three studies. The pooled analysis did not show a significant difference between groups, as evidenced in Fig. 4C (MD –0.41; 95% CI –1.00 to 0.19; *p* = 0.18; I^2^ = 57%; Fig. 4C).

Details on the assessment of plasma and CSF glucose, and HOMA-IR calculations across all included trials are summarized in Supplementary Table 9.

## Discussion

In this systematic review and meta-analysis of eight RCTs including 888 patients, we evaluated the efficacy of intranasal insulin use compared to placebo in adult surgical patients. Our main findings were as follows: (I) intranasal insulin administration was associated with a significant reduction in the incidence of postoperative delirium; (II) there was a significant reduction in IL-6, TNF-α and CRP serum levels; and (III) there were no significant differences in CSF glucose level and HOMA-IR index. TSA further reinforced the robustness of these findings, strengthening the evidence of benefit. Notably, no cases of hypoglycemia were reported across the included studies, underscoring the safety of this intervention in the perioperative setting.

Intranasal insulin bypasses the blood–brain barrier via olfactory and trigeminal pathways, delivering insulin directly to the CNS. It inhibits NF-κB, downregulates proinflammatory cytokines (e.g., TNF-α, IL-1β), modulates the NLRP3 inflammasome, and attenuates glial activation through PI3K/Akt signaling. (Song et al. 2018; Chang et al. 2020; Hennessy et al. 2017). These effects are potentiated by reduced oxidative stress and ferroptosis, and stabilization of neurotransmission lowered glutamate, enhanced GABA, and decreased excitotoxicity. (Yu and Pei 2015) Insulin may also improve cerebral perfusion via NO-mediated vasodilation and enhance metabolism by increasing glucose uptake and astrocytic glycogen (Yu and Pei 2015). Therefore, intranasal insulin has emerged as a promising therapeutic strategy, owing to its capacity to modulate central neuroinflammatory pathways (Huang et al. 2021, 2023; Mi et al. 2023; Yang et al. 2025; Li et al. 2025; Liu et al. 2025b). By attenuating proinflammatory signaling, it may reduce the incidence of delirium, enhance cognitive performance, mitigate neuroinflammation, and improve metabolic homeostasis (Huang et al. 2021, 2023; Mi et al. 2023; Yang et al. 2025; Li et al. 2025; Liu et al. 2025b). After analyzing the included studies, these neuroprotective mechanisms appear consistent with the decreased risk of postoperative delirium observed in our meta-analysis. Most trials enrolled older adults undergoing elective non-cardiac surgery, predominantly abdominal and orthopedic procedures with ASA physical status I–III. Participants were generally free of major neurocognitive disorders at baseline, though considered at risk due to age, comorbidities, or surgical complexity.

Importantly, intranasal insulin administration significantly reduced systemic IL-6 levels, pointing to a possible link between central neuroimmune modulation and peripheral inflammation; CRP concentrations exhibited a parallel and significant decline. Although the reduction in TNF-α levels presented with high heterogeneity and nonetheless remained statistically significant, the magnitude of between-study variability indicates that inflammatory responses may differ across trials, and such heterogeneity warrants cautious interpretation as it may weaken the certainty of conclusions regarding this secondary outcome. These findings bolster the hypothesis that intranasal insulin, while primarily exerting central effects, may also modulate systemic inflammation through downstream mechanisms. One plausible explanation involves the preservation or enhancement of central cholinergic integrity, a critical component of the cholinergic anti-inflammatory pathway (CAP), which mediates systemic immune regulation via vagal efferent signaling (Zivkovic et al. 2015).

This neuroimmune reflex is mediated via efferent vagal signaling and acetylcholine (ACh) release, acting on α7 nicotinic acetylcholine receptors (α7nAChR) expressed by peripheral immune cells, particularly macrophages, thereby suppressing the release of pro-inflammatory cytokines such as TNF-α (Keever et al. 2023). Given intranasal insulin’s capacity to mitigate neuroinflammation, it may help to preserve or restore central cholinergic neuronal integrity, thereby facilitating engagement of the CAP. Notably, cholinergic modulation has also been implicated in the resolution of sepsis-associated cognitive dysfunction and ICU delirium, further underscoring its potential relevance in perioperative neuroimmune regulation (Zivkovic et al. 2015; Neu et al. 2024). While activation of the cholinergic anti-inflammatory pathway represents a biologically plausible mechanism, it should be regarded as one of several possible pathways through which intranasal insulin may exert its systemic immunomodulatory effects. Elucidation of these mechanisms will require dedicated experimental studies integrating central neural circuits with peripheral immune outcomes. Beyond central neuroimmune modulation, alternative mechanisms may also contribute to the observed effects, including differences in pain control, depth of anesthesia, perioperative hemodynamics and postoperative complications (Huang et al. 2021, 2023; Li et al. 2025; Zhang et al. 2016). These factors represent plausible pathways through which intranasal insulin could influence delirium risk, and their potential contribution warrants consideration when interpreting the findings.

Notably, the neuroprotective and cognitive benefits of intranasal insulin remain evident, as corroborated by both clinical and experimental data (Badenes et al. 2021; Yu and Pei 2015; Liu et al. 2025a). Preclinical models of delirium, particularly those employing lipopolysaccharide administration superimposed on neurodegenerative substrates, have shown that peripheral cytokine surges, especially IL-1β and TNF-α can trigger acute cognitive deficits by disrupting cerebral glucose metabolism (Hennessy et al. 2017). These metabolic disturbances disproportionately affect already vulnerable neural networks, compounding the risk of delirium (Kealy et al. 2020). Such findings reinforce the rationale for targeting central inflammatory pathways directly precisely the mechanism by which intranasal insulin is thought to confer benefit. Taken together, these mechanisms substantiate the hypothesis that intranasal insulin mitigates postoperative delirium by preserving cerebral metabolic homeostasis, modulating neuroinflammation, stabilizing neurotransmission and possible contributing to reduction of systemic inflammatory profile via modulation of central cholinergic anti-inflammatory pathway (Kealy et al. 2020, 2015; Maimaiti et al. 2016). This multifaceted central action provides a compelling rationale for its integration into perioperative neuroprotection strategies.

Despite these effects, metabolic markers such as HOMA-IR and serum glucose did not show significant between-group differences, reinforcing the notion that intranasal insulin exerts its benefits without perturbing systemic glucose homeostasis. The Homeostasis Model Assessment of Insulin Resistance (HOMA-IR), calculated from fasting glucose and insulin concentrations, was applied in two included trials (Mi et al. 2023 and Mi et al. 2024) and consistently demonstrated metabolic neutrality. This validates the concept that the neurocognitive benefits of intranasal insulin are mediated primarily through central mechanisms rather than peripheral insulin sensitivity. The absence of systemic effects and hypoglycemia highlights the unique pharmacological profile of intranasal delivery achieving CNS penetration while minimizing peripheral impact and is consistent with prior studies employing even higher intranasal doses (Schmitzberger et al. 2024).Nevertheless, because both HOMA-IR and CSF glucose levels were assessed in only two studies each, these metabolic findings should be interpreted with caution, and additional trials incorporating standardized peripheral markers alongside direct CSF measurements will be essential to more definitively characterize potential central glycemic effects.

Our stratified analysis by intraoperative dexmedetomidine use revealed no difference in the protective effect of intranasal insulin. The effect remained robust across subgroups, suggesting an independent mechanism of action distinct from α2-adrenergic pathways. Dexmedetomidine, while known for its neuroprotective and delirium-preventive properties via α2-receptor agonism, did not modify the efficacy of insulin in this context. (Wang et al. 2018; Duan et al. 2018)

A recent meta-analysis by Zhang et al (Zhang et al. 2025) examined the effect of intranasal insulin on postoperative delirium and reported results broadly concordant with the protective association observed in our study. While the convergence of findings across independent analyses strengthens the credibility of this intervention, our work provides several important and novel methodological contributions that extend beyond the scope of the Zhang review. First, we incorporated one additional randomized controlled trial, thereby expanding the information base and increasing statistical precision. Second, and critically, we applied trial sequential analysis to formally assess whether the accumulated evidence was sufficient to draw reliable conclusions while controlling for the risk of random errors inherent to cumulative meta-analyses. The trial sequential monitoring boundaries were crossed, and the required information size was achieved, indicating that the observed reduction in delirium risk is robust and unlikely to represent a spurious type I error. Moreover, we explicitly addressed a key perioperative confounder that has not been systematically explored in prior syntheses: intraoperative dexmedetomidine exposure. Given the well-established delirium-modulating effects of α₂-agonists, failure to account for this factor may obscure the independent effect of intranasal insulin. Our prespecified subgroup analysis demonstrated a consistent and stable benefit of intranasal insulin irrespective of dexmedetomidine use, with no evidence of effect modification, indicating a mechanism distinct from sedative-mediated neuroprotection. Finally, while three trials originated from the same center, they recruited patients from clearly different surgical settings, including cardiac and non-cardiac procedures, thereby limiting the potential for cohort overlap. Sensitivity analyses excluding these studies produced consistent results, confirmig the robustness of the primary findings. Overall, these methodological refinements extend existing evidence with a more nuanced and statistically rigorous evaluation of intranasal insulin in postoperative delirium prevention.

The optimal dosing and timing of intranasal insulin remain under investigation. Doses of 20–40 IU were well tolerated and associated with lower delirium incidence, with no hypoglycemia reported. This reflects the pharmacological benefit of direct CNS delivery, bypassing systemic glucose regulation. A dose-escalation study showed no hypoglycemia up to 160 IU; events occurred only at 240 IU (Nakadate et al. 2024). Unlike parenteral routes, intranasal insulin achieves rapid CNS absorption with minimal peripheral effects. Future trials with continuous glucose monitoring are warranted to confirm safety across surgical populations. (Liu et al. 2025a) Importantly, the marked variability in dosing strategies, frequency, and timing across existing studies limits the external viability of the current evidence and restricts meaningful comparisons across perioperative settings. Trials using standardized and reproducible dosing protocols are needed to determine the most effective perioperative regimen and to enable more precise clinical recommendations.

Intranasal insulin may be most effective as part of a multimodal delirium prevention strategy. Guidelines recommend targeting multiple etiologies, neuroinflammation, neurotransmitter imbalance, metabolic disruption, pain, infection, and sleep disturbance, especially in high-risk patients (Chaput and Bryson 2012; Skrobik 2011). Given the significant burden of delirium, our observed result underscores its clinical relevance. Future studies should aim to refine patient selection criteria, define optimal dosing and timing strategies, and evaluate the durability of cognitive benefits beyond the immediate postoperative period. Moreover, studies are also needed to further delineate the pathways through which intranasal insulin enhances neuronal resilience and regulates central immune responses.

This study has several important limitations that warrant careful consideration at both the individual study and systematic review levels.

First, the absence of patient level data limited our ability to perform more granular subgroup analyses according to surgical type, baseline comorbidities, perioperative risk profiles, or standardized dosing and timing protocols for intranasal insulin. As a result, potential effect modification by patient or procedure specific factors cannot be fully excluded. Future individual participant data meta-analyses would be particularly valuable to address these questions.Second, the relatively small number of included randomized trials (*n* = 8) constrains the precision of pooled estimates and limits the power to detect modest heterogeneity or publication bias. Although trial sequential analysis suggested that the cumulative evidence reached the required information size for the primary outcome, the overall certainty of evidence remains tempered by the size and scope of the available literature. Third, clinical heterogeneity across studies including variation in surgical populations (cardiac and non-cardiac procedures), intranasal insulin dosing regimens, timing of administration, and baseline delirium risk may have influenced effect estimates. To mitigate this, we conducted predefined subgroup and sensitivity analyses, all of which yielded consistent results; however, residual heterogeneity cannot be entirely excluded. Fourth, although most trials used validated delirium assessment instruments, primarily the Confusion Assessment Method for the ICU (CAM ICU), differences in assessment frequency, timing, and supplementary diagnostic tools may have introduced outcome measurement variability. Such differences could affect delirium detection, particularly for hypoactive or transient presentations. Finally, all included trials were conducted in China, and several originated from the same research center. Although these studies enrolled distinct surgical populations, this concentration may limit external validity and raises concerns regarding generalizability to other healthcare systems, ethnic backgrounds, and perioperative practices. Accordingly, the findings should be interpreted within this contextual framework. Certainty of evidence was mainly downgraded for imprecision related to the small number of trials and for indirectness due to geographic and population concentration. Despite supportive trial sequential analysis, residual clinical heterogeneity limits overall certainty and underscores the need for larger multicenter studies.

Taken together, these limitations underscore the need for future large scale, multicenter randomized trials conducted across diverse geographic regions and healthcare settings, with standardized intervention protocols and delirium assessment strategies. Such studies are essential to confirm generalizability, refine patient selection, and define the role of intranasal insulin within multimodal delirium prevention pathways.

## Conclusion

This systematic review and meta-analysis found that preoperative intranasal insulin significantly reduced the incidence of postoperative delirium and decreased systemic inflammatory markers (IL-6, CRP, and TNF-α) without affecting glycemic stability. These results underscore its potential as a safe, CNS-targeted strategy for perioperative neuroprotection. However, while intranasal insulin appears effective in reducing the incidence of delirium, it has not yet been shown to mitigate the consequences or complications of delirium itself, which remain the major determinants of patient outcomes. Further research is needed to clarify mechanisms, optimize administration protocols, and assess both long-term cognitive outcomes and the broader clinical impact of this intervention.

## Supplementary Information

Supplementary Material 1.

## Data Availability

The data underlying this article are available in the article and in its online supplementary material.

## Abbreviations

3D-CAM: 3-Minute Diagnostic Assessment for CAM-defined delirium
α7nAChR: α7 Nicotinic acetylcholine receptor
ACh: Acetylcholine
ASA: American Society of Anesthesiologists
BMI: Body Mass Index
CAM-ICU: Confusion Assessment Method for the Intensive Care Unit
CAP: Cholinergic Anti-inflammatory Pathway
CNS: Central Nervous System
CRP: C-Reactive Protein
CSF: Cerebrospinal Fluid
DRS: Revised Delirium Rating Scale
HOMA-IR: Homeostatic Model Assessment of Insulin Resistance
IL-6: Interleukin-6
ISPOCD: International Study of Postoperative Cognitive Dysfunction
IU/U: International Units / Units
MD: Mean Difference
MMSE: Mini-Mental State Examination
N/A: Not Available / Not Applicable
NF-κB: Nuclear Factor kappa B
OR: Odds Ratio
POD: Postoperative Delirium
PRISMA: Preferred Reporting Items for Systematic Reviews and Meta-Analyses
RCT: Randomized Controlled Trial
RIS: Required Information Size
RoB-2: Risk of Bias 2
SD: Standard Deviation
TSA: Trial Sequential Analysis
TNF-α: Tumor Necrosis Factor-alpha
