# Supplementary material for: Effect of preoperative intranasal insulin on postoperative delirium in adult surgical patients: a systematic review, meta-analysis, and trial sequential analysis
Source: Perioper Med (Lond). 2026 Mar 20;15:33. doi: 10.1186/s13741-026-00674-5 (PMC13063771; doi:10.1186/s13741-026-00674-5)
Supplement: Supplementary file 1 — Supplementary Material 1. [file 13741_2026_674_MOESM1_ESM.docx]

**Supplementary Material**

**Supplementary Table 1**. Complete search strategy.

**Supplementary Figure 1.** Critical appraisal of studies according to Cochrane Collaboration’s Risk of Bias 2 (RoB-2) tool

**Supplementary Figure 2.** Funnel plot for postoperative delirium

**Supplementary Figure 3.** Leave-one-out analysis for postoperative delirium

**Supplementary Figure 4.** Trial sequential analysis (TSA) of the effect of intranasal insulin on postoperative delirium.

**Supplementary Figure 5.** Forest plot of the effect of preoperative intranasal insulin on postoperative delirium including all published trials (Huang et al. 2021, 2023, 2024).

**Supplementary Figure 6.** Forest plot of the effect of preoperative intranasal insulin on postoperative delirium after exclusion of the three Huang et al. trials.

**Supplementary Table 7.** Methodological details of biospecimen collection, assays, and delirium assessments across included trials

**Supplementary Figure 8** Risk ratios for postoperative delirium stratified by studies using validated CAM/CAM-ICU tools versus those employing non-CAM neuropsychological assessments (OTHER subgroup).

**Supplementary Table 9.** Glucose metabolism and HOMA-IR assessments across included trials

**Supplementary Table 1.** Complete Search Strategy

Search: **("intranasal insulin" OR "central insulin administration") AND (delirium OR "postoperative cognitive dysfunction" OR "perioperative neurocognitive disorder" OR cognitive OR memory)**

("intranasal insulin"[All Fields] OR "central insulin administration"[All Fields]) AND ("delirium"[MeSH Terms] OR "delirium"[All Fields] OR "delirium s"[All Fields] OR "deliriums"[All Fields] OR "postoperative cognitive dysfunction"[All Fields] OR "perioperative neurocognitive disorder"[All Fields] OR ("cognition"[MeSH Terms] OR "cognition"[All Fields] OR "cognitions"[All Fields] OR "cognitive"[All Fields] OR "cognitively"[All Fields] OR "cognitives"[All Fields]) OR ("memories"[All Fields] OR "memory"[MeSH Terms] OR "memory"[All Fields] OR "memory s"[All Fields]))

**Translations**

**delirium:** "delirium"[MeSH Terms] OR "delirium"[All Fields] OR "delirium's"[All Fields] OR "deliriums"[All Fields]

**cognitive:** "cognition"[MeSH Terms] OR "cognition"[All Fields] OR "cognitions"[All Fields] OR "cognitive"[All Fields] OR "cognitively"[All Fields] OR "cognitives"[All Fields]

**memory:** "memories"[All Fields] OR "memory"[MeSH Terms] OR "memory"[All Fields] OR "memory's"[All Fields]


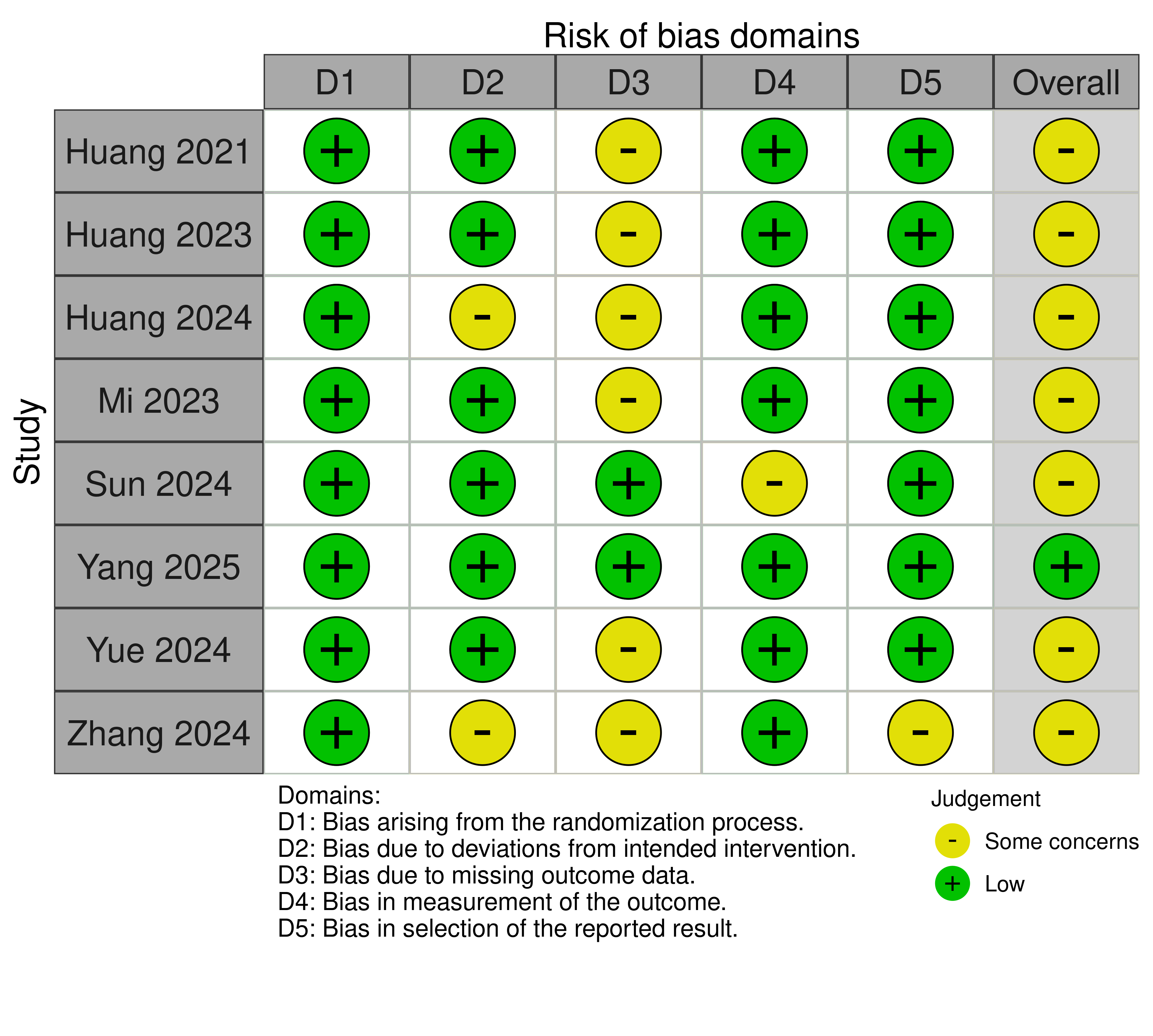


**Supplementary Figure 1.** Critical appraisal of studies according to Cochrane Collaboration’s Risk of Bias 2 (RoB-2) tool

**Supplementary Figure 2.** Funnel plot for the primary outcome


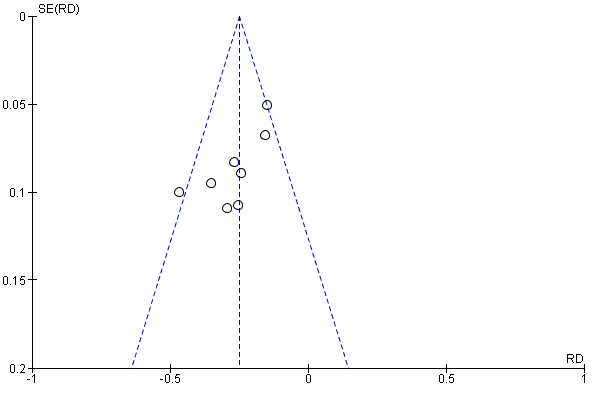
**
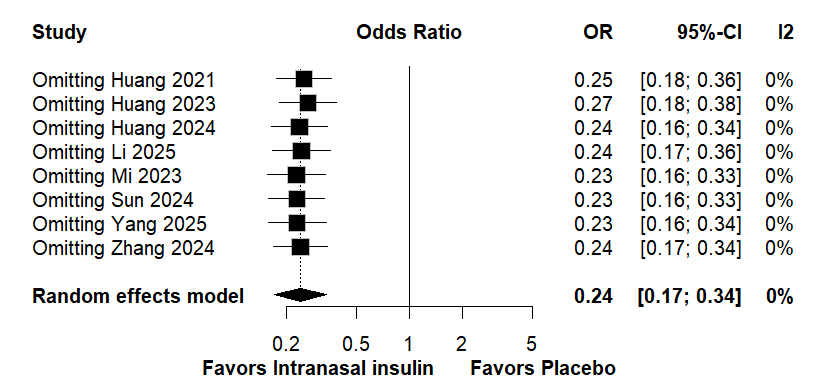
Supplementary Figure 3.** Leave-one-out analysis for the primary outcome

**Supplementary Figure 4.** Trial sequential analysis (TSA) of the effect of intranasal insulin on postoperative delirium.


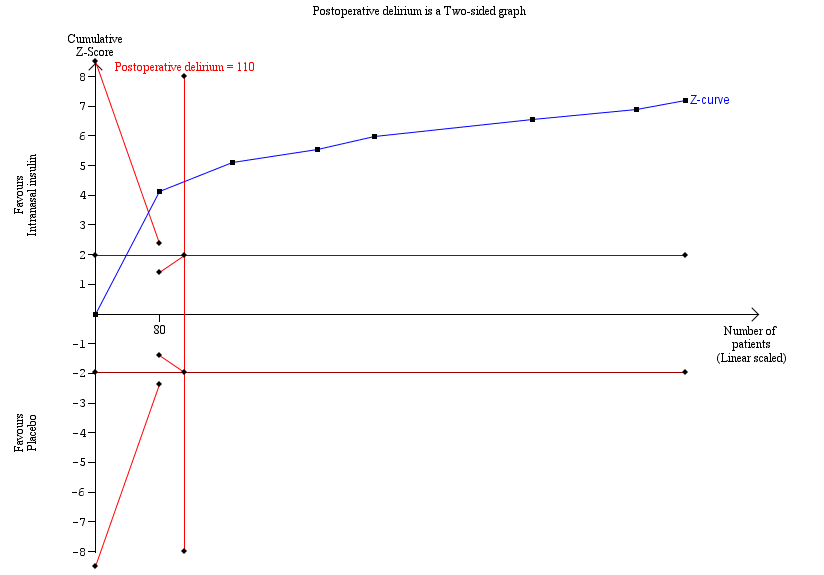


**Supplementary Figure 5.** Forest plot of the effect of preoperative intranasal insulin on postoperative delirium including all published trials (Huang et al. 2021, 2023, 2024).


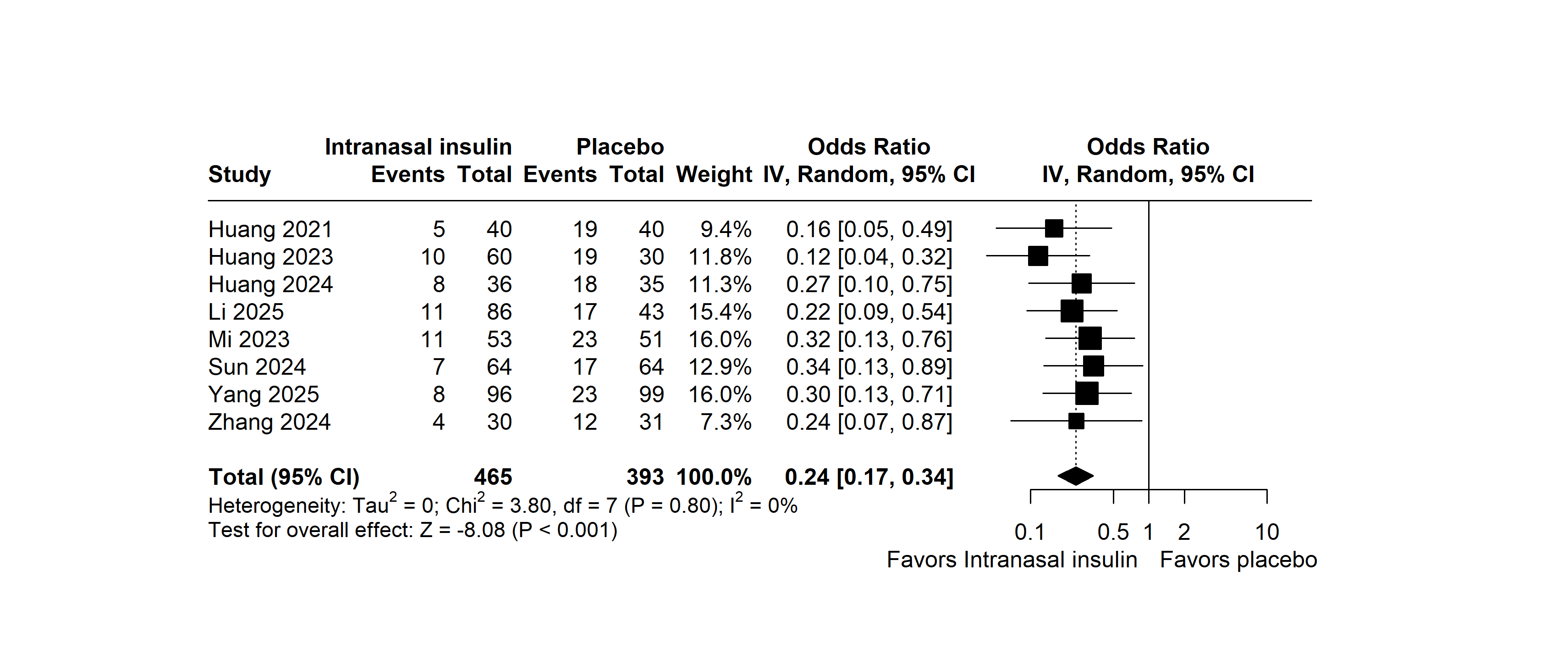


**Supplementary Figure 6.** Forest plot of the effect of preoperative intranasal insulin on postoperative delirium after exclusion of the three Huang et al. trials, which were suspected to derive from overlapping patient datasets.

**
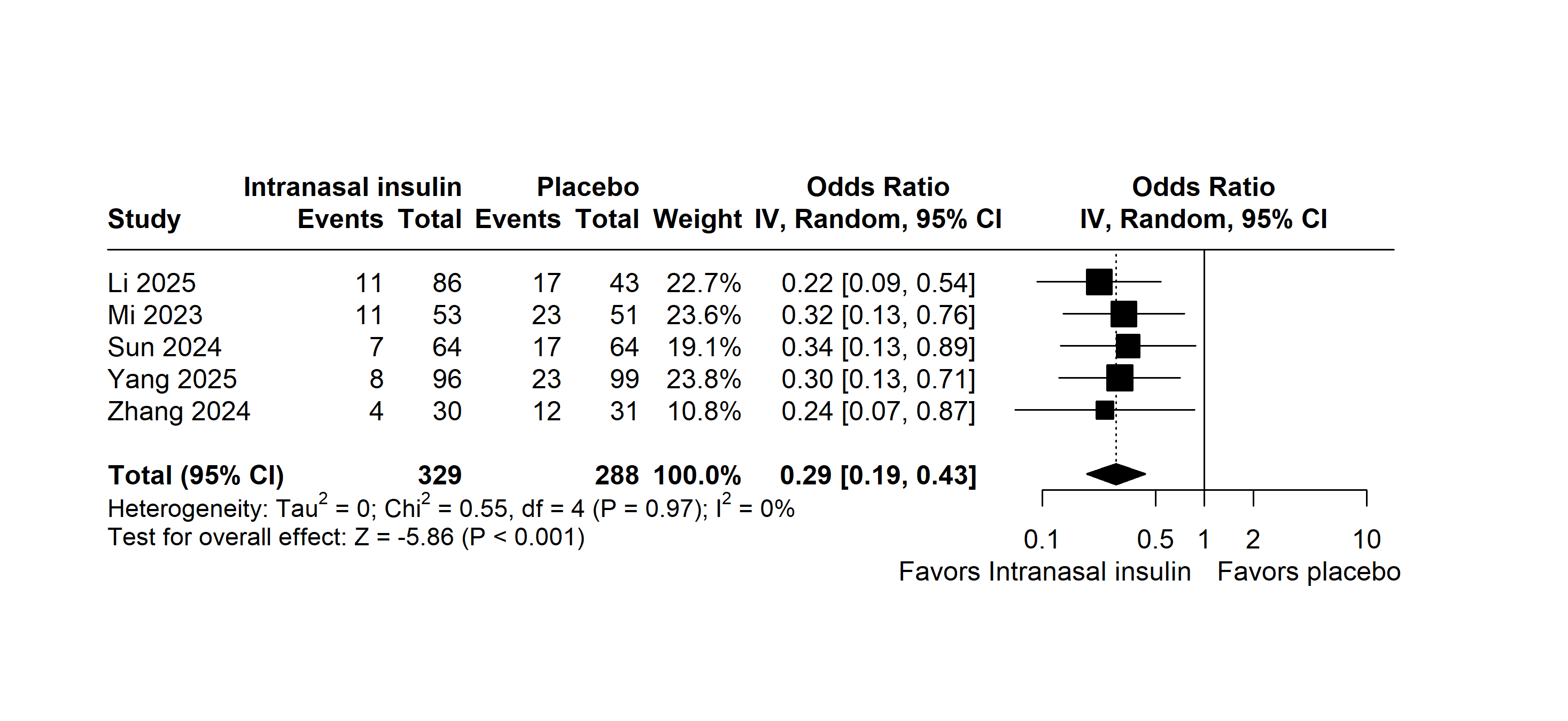
**

**Supplementary Table 7.** Methodological details of biospecimen collection, assays, and delirium assessments across included trials

| **Study (first author, year)** | **Sample type(s)** | **Timing of collection** | **Processing & storage** | **Assay method / location** | **Delirium assessment (instrument, timing)** | **Preoperative delirium screening** |
| --- | --- | --- | --- | --- | --- | --- |
| **Huang et al. 2021** | Jugular venous blood (3 mL) | Before first dose, end of surgery, POD1, POD3, POD5 | Centrifuged 4000 rpm 10 min; serum frozen −80°C | ELISA (FANKEW, Shanghai); site not specified | RASS → CAM-ICU once daily POD1, 3, 5 | Not reported |
| **Huang et al.2023** | Venous blood (3 mL) | Before insulin/saline, end of surgery, POD1, POD2, POD3 | Centrifuged 3000 g 15 min; serum stored −80°C | ELISA (Thermo Fisher, US); blinded staff | RASS → CAM-ICU twice daily (08–10h, 18–20h), POD1–3 | Yes, via MMSE; patients with cognitive impairment excluded |
| **Huang et al. 2024** | Venous blood | Pre-intervention, 1 d before surgery, before induction | Serum stored −80°C | Cortisol by ELISA (Shanghai Fanke Biotech) | RASS → CAM-ICU and CAM-CR twice daily, POD1–3 | Pre-op MMSE performed |
| **Mi et al. 2023** | Venous blood | Day of surgery, morning POD1, POD3, POD7 | Central lab; methods not fully described | ELISA / immunoturbidimetry depending on marker | No delirium (study on POCD with neuropsychological batteries) | Pre-op MMSE ≥24 |
| **Yang et al. 2025** | Venous blood, CSF (1 mL during spinal) | Serum: baseline, POD1, POD3; CSF: intraop before anesthetic | Frozen immediately at −80°C | ELISA kits (OC, ucOC, insulin); glucose oxidase for glucose | RASS → CAM-ICU twice daily (08–10h, 19–21h), POD1–5; severity by DRS-R-98 | Not reported |
| **Sun et al. 2024** | Venous blood | POD1 | Not described | Serum IL-6, TNF-α, S100β, CRP (methods not specified) | 3D-CAM daily POD1–3 | Not specified |
| **Yue Li et al.2025** | CSF (1 mL), fingertip blood | CSF: before spinal anesthetic; fingertip glucose periop; CSF lactate/glucose | Not specified | Biochemistry (methods not specified) | RASS → CAM-ICU daily 18–19h, POD1–3 | Yes, delirium pre-op was exclusion |
| **Zhang et al. 2024** | (No delirium assessment; biomarker only) | Blood sampling reported, timing not linked to POD | Processing: centrifuged and frozen (−80°C) | Assays: described (likely ELISA or biochemical kits) | ISPOCD – study eligible for acute POD | Not applicable |

**Supplementary Table 8** Risk ratios for postoperative delirium stratified by studies using validated CAM/CAM-ICU tools versus those employing non-CAM neuropsychological assessments (OTHER subgroup).

**
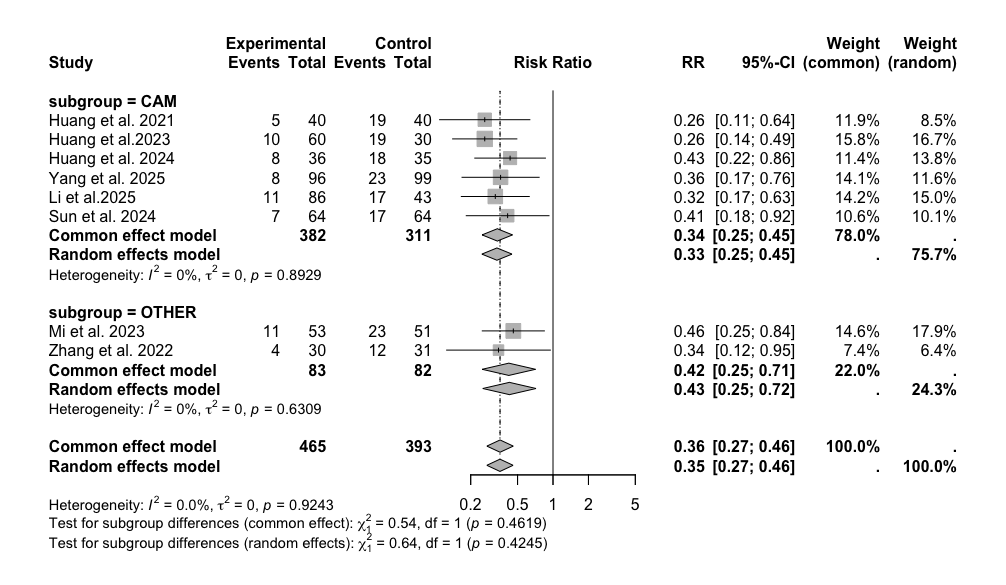
** *Legend:The “OTHER” subgroup comprises trials that did not use CAM or CAM-ICU to diagnose postoperative delirium. Zhang et al. 2024 applied an ISPOCD-based neuropsychological battery and reported biomarker changes without delirium assessment. Mi et al. 2023 likewise evaluated postoperative cognitive dysfunction using structured neuropsychological testing rather than CAM-based instruments. These studies were retained for completeness but analyzed separately due to their distinct outcome assessment methods****.***

# Supplementary Table 9. Glucose metabolism and HOMA-IR assessments across included trials

| Study (year) | Plasma glucose | CSF glucose | Insulin measured | HOMA-IR |
| --- | --- | --- | --- | --- |
| Huang 2021 | Yes | No | Yes | No |
| Huang 2023 | Yes | No | Yes | No |
| Mi 2023 | Yes | No | Yes | Yes |
| Sun 2024 | Yes | No | No | Yes |
| Yue Li 2025 | No | Yes | No | No |
| Yang 2025 | No | Yes | Yes | Yes |

**Glucose serum levels**

Serum glucose was monitored using heterogeneous methodologies across the included trials. In Huang (2021), venous glucose was recorded at baseline and postoperatively solely for safety surveillance of hypoglycemia, without specification of the analytical technique. In Huang (2024), a continuous glucose monitoring system provided dynamic perioperative profiles, including mean glucose values and hypoglycemic episodes. Sun (2024) reported perioperative fasting plasma glucose together with insulin and HOMA-IR indices, although the precise laboratory assay was not described. Mi (2023) performed serial fasting venous sampling (baseline, postoperative days 1, 3, and 7), with glucose quantified in the central laboratory using a turbidimetric inhibition immunoassay, insulin measured by ELISA, and HOMA-IR calculated accordingly. Yue Li (2025) employed fingertip capillary testing at defined perioperative time points (40 minutes after intervention, at operating room entry, and at procedure completion) and additionally obtained cerebrospinal fluid samples for glucose and lactate measurement.

**Glucose CSF levels**

Cerebrospinal fluid (CSF) glucose was assessed in two trials. In the study by Yang et al. (2025), a small volume of CSF was obtained during induction of spinal anesthesia immediately after dural puncture and before intrathecal drug administration; samples were processed by centrifugation, stored at –80 °C, and analyzed using a glucose oxidase assay kit in parallel with ELISA-based measurements of insulin and osteocalcin. In the trial by Yue Li et al. (2025), 1 mL of CSF was aspirated prior to the administration of local anesthetic during spinal anesthesia for hip fracture surgery and subsequently analyzed for glucose and lactate levels to evaluate the central metabolic effects of intranasal insulin.

**HOMA-IR.**

Insulin resistance was uniformly assessed using the homeostasis model assessment of insulin resistance (HOMA-IR), calculated as fasting insulin × fasting glucose / 22.5. In Mi et al. (2023), venous blood samples were obtained preoperatively and on postoperative days 1, 3, and 7; glucose was measured in the hospital’s central laboratory by turbidimetric inhibition immunoassay, and insulin was quantified by ELISA. Sun et al. (2024) measured fasting glucose and insulin at baseline, intraoperatively, and on the first postoperative day, with HOMA-IR computed at each time point. In the trial by Yang et al. (2025), peripheral plasma was collected at baseline, day 0, day 1, and day 3; fasting glucose was determined with a glucose oxidase assay kit, insulin by solid-phase ELISA, and HOMA-IR calculated accordingly, with predefined cutoffs for insulin resistance. Across these studies, HOMA-IR served as a surrogate marker of perioperative insulin sensitivity, enabling comparison of metabolic changes associated with intranasal insulin administration.
